# Comparison of four pharmacological strategies aimed to prevent the lung inflammation and paraquat-induced alveolar damage

**DOI:** 10.1186/s13104-019-4598-0

**Published:** 2019-09-18

**Authors:** Jefferson Antonio Buendía, José Armando Justinico Castro, Laura Joanna Tapia Vela, Denis Sinisterra, Juana Patricia Sánchez Villamil, Andrés Felipe Zuluaga Salazar

**Affiliations:** 1 Grupo de Investigación en Farmacología y Toxicología, Centro de Información y Estudio de Medicamentos y Tóxicos (CIEMTO), Facultad de Medicina, Universidad de Antioquia, Carrera 51D #62-29, Medellín, Colombia; 2 Departamento de Patología, Facultad de Medicina, Universidad de Antioquia, Medellín, Colombia; 3 Facultad de Odontología, Sede Bucaramanga, Universidad Antonio Nariño, Bogotá, Colombia

**Keywords:** Paraquat, Inflammation, Lung

## Abstract

**Objective:**

The aim of this study was to compare in vivo effect of five pharmacological options on inflammation and pulmonary fibrosis induced by paraquat.

**Methods:**

54 Wistar SPF rats were used. After 2 h post-intoxication with paraquat ion, groups of 9 animals were randomly assigned to (1) cyclophosphamide plus dexamethasone (2) low molecular weight heparin (3) unfractionated heparin (4) vitamin C every 24 h, (5) atorvastatin or (6) placebo with intraperitoneal saline. Lung inflammation, alveolar injury, hepatocyte damage, hepatic regeneration, acute tubular necrosis and kidney congestion were evaluated.

**Results:**

In the control group 100% of animals presented moderate and severe lung inflammation, while in the groups with atorvastatin and intratracheal heparin this proportion was lower (55.5%; CI 26.6–81.3%) (p = 0.025). A lower degree of moderate or severe hepatic regeneration was evident in the treatment groups with atorvastatin (p = 0.009). In this study was demonstrated that statins and heparin might have a protective effect in the paraquat-induced destructive phase. More evidence is needed to evaluated of dose–response effects of these drugs before to study in clinical trials.

## Introduction

Paraquat (PQ) poisoning is a major health problem worldwide, mainly due to self-poisoning related with suicides or by occupational exposure [1]. Globally, 250,000 to 370,000 people die from pesticide poisoning each year, and more than 90% of the individuals with acute poisoning attempted to commit suicide by intentionally ingesting PQ [2, 3]. Most deaths occur in Southeast Asia, Central and South America [1]. The marketing of PQ has been banned in 32 countries, but its low-cost and unrestricted availability promotes its extensive use mainly in rural areas in developing countries [4, 5]. Acute respiratory failure and pulmonary fibrosis are the main causes of death by PQ intoxication [6]. There is no effective treatment to prevent acute respiratory failure or the development of pulmonary fibrosis [6, 7]. The current evidence is limited to cyclophosphamide and glucocorticoids; which have a modest effect in these outcomes [7, 8]. The lung injury seems to be due to the massive generation of reactive oxygen species such as: superoxide anion, hydroxyl, peroxide radicals by the PQ in the lung parenchyma; which quickly overcome any antioxidant defense [9]. The search of substances aimed to prevent the lung injury include chelators that reduce the degree of cellular exposure to PQ (such as heparin) [10] or drugs with antioxidant action to restore the cellular effect of PQ (such as vitamin C, statins) [11, 12]. However, there are no head-to-head comparisons between these promising substances, and their effects have not been evaluated in other organs injured by PQ intoxication such as liver and kidney. It is necessary to have better therapeutic options to treat PQ poisoning, but controlled clinical studies have important ethical limitations in this field; therefore animal models can be an interesting alternative to this limitation. Our objective was to compare the in vivo effect of five pharmacological options on inflammation, alveolar damage, and pulmonary fibrosis induced by PQ.

## Main text

### Animals

54 Wistar SPF rats (50% female) of 8 weeks with weights of 200 ± 10 g from the University of Antioquia biotherium were used. The rats (our experimental unit) were, housed with a maximum density of 3 animals per box, with access to concentrated feed and water at will, temperature between 20 °C and 25 °C, under controlled light–dark conditions. Ethical aspects: the study was approved by the Animal Experimentation Committee of the University of Antioquia (1-2025).

### Animal model of toxicity

In the laboratory, all animals received 30 mg/kg of paraquat ion by oral probe, this dose is within the range of lethal dose 50 described for rats (http://pmep.cce.cornell.edu/profiles/extoxnet/metiram-propoxur/paraquat-ext.html#10). We estimated a sample size of 9 rats per group to detect minimum differences of 50% in the degree of acute inflammation between the placebo group and each of the experimental groups, assuming an alpha error of 5% and a power of 80%. After 2 h post-intoxication, groups of 9 animals were randomly assigned (using a sequence of random numbers generated by STATA^®^) in one of the following six experimental arms: (1) cyclophosphamide 15 mg/kg plus dexamethasone 30 mg/kg (single doses), (2) low molecular weight heparin 60, 80 or 100 U/kg every 12 h subcutaneously for 2 days (3 animals per dose), (3) unfractionated heparin (UFH) 5, 25 or 50 mg/kg every 24 h intratracheal (3 animals per dose) for 2 days, (4) vitamin C 20, 40 or 60 mg/kg every 24 h (3 animals per dose) for 3 days, (5) atorvastatin 10, 20, or 40 mg/kg every 24 h (3 animals per dose) for 8 days, or (6) placebo with intraperitoneal saline (single doses). Health conditions were evaluated every 3 h for up to 21 days while the rats survived. All experimental groups were treated and assessed at the same time.

### Histopathological analysis

At the end of the treatment, euthanasia for cervical dislocation was performed under anesthesia with isoflurane (Abbott, USA). All evaluators were blinded to the assigned treatment. The extracted tissues were placed in a 10% formalin solution and embedded in paraffin to make sections with the 4-micron microtome. The tissues were initially colored with hematoxylin and eosin for evaluation under conventional optical microscopy. In the lung, Masson’s trichrome staining samples were performed to quantify collagen deposition. In the liver, trichrome staining, PAS, PAS diastase and cytokeratin 7 were used to assess bile duct integrity and CD68 to quantify Kupffer cells. In the kidney, trichrome stain, kidney methenamine silver, Masson fontanel, and PAS were used to evaluate the glomerular basement membrane and quantify the presence of hyaline globules in renal tubules. The histopathological evaluation of all the samples was carried out by a group of three pathologists and two pathology residents, when there was disagreement in any of the cases, the concept of an additional expert was requested.

### Histopathological evaluation in lung

Lung inflammation was evaluated in the interstice and airway. The degree of interstitial inflammation (presence inflammatory infiltrate) was classified in three categories: mild inflammation (inflammatory compromise < 25% in the sample analyzed), moderate (inflammatory compromise of 25 to 50% in the sample analyzed), and severe (inflammatory compromise > 50% in the sample analyzed). The degree of alveolar injury was evaluated in three categories: severe alveolar injury was considered if there was the destruction of alveolar architecture with hemorrhage and edema. The moderate alveolar injury was considered if there were only one of these two findings, and mild alveolar injury was considered when alveolar architecture was preserved and there was absence of hemorrhage and edema. Interstitial fibrosis was evaluated under the Aschroft scale. A normal lung or minimal wall thickening without architectural damage was considered mild fibrosis. Moderate wall thickening of defined lung structure or fibrosis with defined lung structure damage were considered as moderate fibrosis, and severe distortion of fibrotic structures or total fibrous obliteration were considered as severe fibrosis.

### Histopathological evaluation in liver and kidney

Histopathological changes in liver and kidney were evaluated in 33 rats. The hepatocyte damage was evaluated according to the presence or absence of liquefactive or ischemic necrosis foci. To classify hepatic regeneration the variables of ballooning, binucleation and increased mitosis in hepatocytes were evaluated. With three characteristics fulfilled it was considered as severe regeneration if it presented only two were considered as moderate regeneration and mild with only one of them. Other histopathological variables evaluated were ductopenia, stage of destruction of the ductal epithelium, increase of Kupffer cells, fibrosis and congestion. In the kidneys, acute tubular necrosis and kidney congestion were evaluated qualitatively as present or absent.

### Statistical analysis

The unit of analysis was a single animal. Fisher’s exact test was used to compare frequencies of number of rats by histopathological findings between treatments. Values of p less than or equal to 0.05 were considered as statistical significance in the hypothesis tests.

### Results

**Fig. 1:**
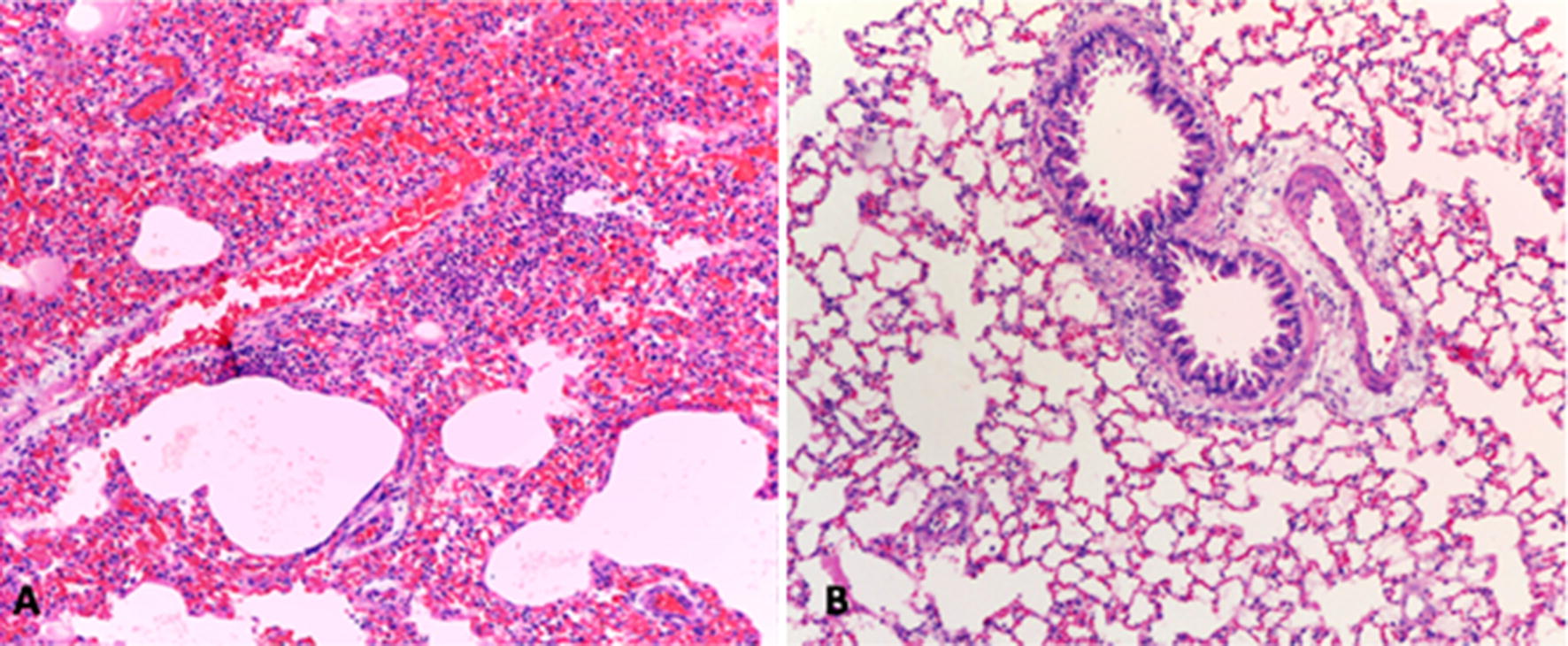
**a** Rat lung intoxicated with PQ without treatment: presents important interstitial mononuclear inflammatory infiltrate concomitant with intra-alveolar hemorrhage (H&E, ×40). **b** Rat lung intoxicated with PQ and treated with intratracheal heparin: conserved alveolar architecture, without hemorrhage or inflammatory infiltrate (H&E, ×40)

There are not differences in measures of lung morphometry (weight and length) by treatments, see Additional files 1, 2. In the control group with PQ, 100% of rats presented moderate and severe lung inflammation, while in the groups treated with atorvastatin and intratracheal heparin this proportion was lower (55.5%; CI 26.6–81.3%) (p = 0.025) see Table 1, Fig. 1. A lower degree of moderate or severe hepatic regeneration was evident in the treatment groups with atorvastatin (20%; CI 10.5–70.1%) and subcutaneous heparin (0%) with respect to the control group with PQ (62.5%; CI 30.5–86.3%) (p = 0.009), see Table 2.

**Table 1:** Number of rats by lung histopathological findings between treatments

Variable	Severity	Cicl/Dex	Ator	Vit C	HepSC	HepIT	PQ	p
		n = 9	n = 9	n = 9	n = 9	n = 9	n = 9	
Alveolar injury	Absent/mild	4	3	1	2	2	1	0.5275
	Moderate/severe	5	6	8	7	7	8	
Lung inflammation	Absent/mild	1	4	0	1	4	0	0.2699
	Moderate/severe	8	5	9	8	5	9	
Interstitial fibrosis	Absent/mild	7	9	9	7	8	9	0.2699
	Moderate/severe	2	0	0	2	1	0	

*Cicl/Dex* cyclophosphamide–dexamethasone, *Ator* atorvastatin, *Vit C* vitamin C, *HepSC* low molecular weight heparin, *HepIT* unfractionated heparin intratracheal, *PQ* paraquat

**Table 2:** Number of rats by liver and renal histopathological findings between treatments

Variable	Severity	Cicl/Dex	Ator	Vit C	HepSC	HepIT	PQ	p
		n = 5	n = 5	n = 4	n = 6	n = 4	n = 8	
Hepatocyte damage	Absent	0	1	3	4	3	2	0.057
	Present	5	4	1	2	1	6	
Hepatic regeneration	Mild	1	4	0	5	1	3	*0.009*
	Moderate	2	0	4	1	3	1	
	Severe	2	1	0	0	0	4	
Acute tubular necrosis	Absent	2	2	2	4	2	3	0.493
	Present	3	0	1	2	3	6	
Kidney congestion	Absent	5	2	3	6	4	5	0.067
	Present	0	0	0	0	0	4	

*Cicl/Dex* cyclophosphamide–dexamethasone, *Ator* atorvastatin, *Vit C* vitamin C, *HepSC* low molecular weight heparin, *HepIT* unfractionated heparin intratracheal, *PQ* paraquat

### Discussion

This is the first head-to-head comparison between promising substances to prevent acute consequences of PQ intoxication. To the best of our knowledge, none of these strategies had been compared experimentally against the standard strategy of cyclophosphamide and glucocorticoids. Atorvastatin and heparin have a relevant effect to reduce the degree of lung inflammation and alveolar injury during PQ intoxication. Respect to atorvastatin our findings are similar to published by Khodayar et al. in 2014 [13]. In this study elevated levels of hydroxyproline and malondialdehyde,—tissue markers of inflammation—induced by PQ were attenuated significantly by atorvastatin at the doses of 10, 20 and 40 mg/kg. This effect can be explained by their actions in PPARs receptors and the regulation it exerts on the production of nitric oxide synthase, leukotriene B and platelet-activating factor [14–16]. Respect to heparin, our findings are similar than described by Liu and Jian [10]. This study found a decrease in levels of hydroxyproline compared with the control group without treatment. The promising protective effects of heparin on both lung and liver may be related with two mechanisms: the uptake of positively charged molecules due to its polyanion properties, the effect on xanthine oxidase by decreasing and regulating the concentration of this enzyme which reduces the production of reactive oxygen species in the endothelium [17–21].

The experimental arm that received vitamin C showed less hepatocyte damage, with similar histological patterns described by Awadalla in 2012 [22]. In this study Vitamin C administration attenuated the morphological damages induced by PQ in the liver of experimental animals; being these results were attributed to vitamin C’s ability to capture free radicals from paraquat-induced stress and anti-apoptosis activity. Preclinical studies in liver injury show that the benefits of Vitamin C were principally implicated in regulating signaling pathways, such as inflammation-associated TNF signaling pathway, NF-κB signaling pathway [23, 24]. These studies give arguments to evaluate the protective role against acute liver injury that can occur in patients with acute intoxication especially in those with high doses of ingestion which have the highest risk of developing acute liver disease and placing the patient’s life at risk.

## Limitations

This is an exploratory study, and among our limitations are first the small samples of sizes, which despite this was able to show statistically significant differences as mentioned above. Although we found histopathological evidence of a reduction in the degree of inflammation with some drugs, to have greater certainty of said changes, it is necessary to evaluate the alveolar epithelial injury need at the molecular and biochemical level to confirm these results, so our finding is preliminary and not conclusive about this effect. However, the authors believe that our results are relevant and motivating to continue the study of these drugs in pulmonary fibrosis induced by PQ. Likewise, the use of semi-quantitative scales which we had to design due to the absence of these in the literature may not make the results comparable with other studies; however, the histopathological criteria described for their design are part of the routine description at pathological level facilitating their understanding. Finally, the generation of fibrosis is the most important sequel in survivors of paraquat poisoning; our study focused on evaluating acute outcomes (inflammation and organ damage) so that fibrosis is not the essential outcome to compare, this may reduce the ability to extrapolate results to humans. According to reports in the literature, it takes at least 7 days to develop fibrosis [6].

## Supplementary information

**Additional file 1.** Baseline data for each experimental group.

**Additional file 2.** Measures of lung morphometry between treatments.

## Data Availability

The raw data supporting your findings can request to CIEMTO-UdeA (http://ciemto.medicinaudea.co/).

## Abbreviations

PQ: paraquat
UFH: unfractionated heparin
