# Supplementary material for: Comparison of four pharmacological strategies aimed to prevent the lung inflammation and paraquat-induced alveolar damage
Source: BMC Res Notes. 2019 Sep 18;12:584. doi: 10.1186/s13104-019-4598-0 (PMC6749662; doi:10.1186/s13104-019-4598-0)
Supplement: Supplementary file 1 — Additional file 1. Baseline data for each experimental group. [file 13104_2019_4598_MOESM1_ESM.docx]

| **Variable, median (IQR)** | **PQ** | **Cicl/Dex** | **Ator** | **Vit C** | **Hep SC** | **Hep IT** | **p** |
| --- | --- | --- | --- | --- | --- | --- | --- |
| **Weight (grams)** | 492.9(4) | 492.3 (45.29) | 481.8 (222.6) | 497.9 (265.2) | 460.3 (40.29) | 484.4 (33.5) | 0,081 |
| **Deaths, n(%)** | 8(88,8) | 8(88,8) | 9(100) | 9(100) | 6(66,6) | 7(77,7) | 0,372 |
| **Median survival (hours)** | 3(0) | 2(1) | 2(0) | 3(1) | 3(0) | 2(1) | 0.0713 |
|  |  |  |  |  |  |  |  |

**Additional Material**

**Baseline data for each experimental group**

Cicl/Dex: cyclophosphamide – dexamethasone, Ator: atorvastatin, Vit C: Vitamin C, HepSC: low molecular weight heparin, HepIT: unfractionated heparin intratracheal PQ: Paraquat. interquartile range (IQR)
