# Supplementary material for: Comparison of four pharmacological strategies aimed to prevent the lung inflammation and paraquat-induced alveolar damage
Source: BMC Res Notes. 2019 Sep 18;12:584. doi: 10.1186/s13104-019-4598-0 (PMC6749662; doi:10.1186/s13104-019-4598-0)
Supplement: Supplementary file 2 — Additional file 2. Measures of lung morphometry between treatments. [file 13104_2019_4598_MOESM2_ESM.docx]

**Additional Material**. **Measures of lung morphometry** **between treatments.**

| **Variable** | **Cicl/Dex** | **Ator** | **Vit C** | **HepIP** | **HepIT** | **PQ** | **p** |
| --- | --- | --- | --- | --- | --- | --- | --- |
| Weight (grs) | 3,60 | 4,20 | 3,90 | 3,90 | 3,90 | 4,05 | 0.152 |
| Length (cm) | 2,60 | 2,60 | 2,50 | 2,60 | 2,65 | 2,50 | **0,65** |

Cicl/Dex: cyclophosphamide – dexamethasone, Ator: atorvastatin, Vit C: Vitamin C, HepSC: low molecular weight heparin, HepIT: unfractionated heparin intratracheal PQ: Paraquat
